# Preparation, Biosafety, and Cytotoxicity Studies of a Newly Tumor-Microenvironment-Responsive Biodegradable Mesoporous Silica Nanosystem Based on Multimodal and Synergistic Treatment

**DOI:** 10.1155/2020/7152173

**Published:** 2020-11-05

**Authors:** Zelai He, Huijun Zhang, Hongwei Li, Yanyan Wang, Jing Qian, Xixi Cai, Li Sun, Jingwen Huang

**Affiliations:** ^1^The First Affiliated Hospital of Bengbu Medical College & Tumor Hospital Affiliated to Bengbu Medical College, Bengbu 233004, China; ^2^Department of Cardiothoracic Surgery, Huashan Hospital of Fudan University, Shanghai 200040, China; ^3^The Second Affiliated Hospital of Bengbu Medical College, Bengbu 233004, China

## Abstract

Patients with triple negative breast cancer (TNBC) often suffer relapse, and clinical improvements offered by radiotherapy and chemotherapy are modest. Although targeted therapy and immunotherapy have been a topic of significant research in recent years, scientific developments have not yet translated to significant improvements for patients with TNBC. In view of these current clinical treatment shortcomings, we designed a silica nanosystem (SNS) with Nano-Ag as the core and a complex of MnO_2_ and doxorubicin (Dox) as the surrounding mesoporous silica shell. This system was coated with anti-PD-L1 to target the PD-L1 receptor, which is highly expressed on the surface of tumor cells. MnO_2_ itself has been shown to act as chemodynamic therapy (CDT), and Dox is cytotoxic. Thus, the full SNS system presents a multimodal, potentially synergistic strategy for the treatment of TNBC. Given potential interest in the clinical translation of SNS, the biological safety and antitumor activity of SNS were evaluated in a series of studies that included physicochemical characterization, particle stability, blood compatibility, and cytotoxicity. We found that the particle size and zeta potential of SNS were 94.6 nm and -22.1 mV, respectively. Ultraviolet spectrum analysis showed that Nano-Ag, Dox, and MnO_2_ were successfully loaded into SNS, and the drug loading ratio of Dox was about 10.2%. Stability studies found that the particle size of SNS did not change in different solutions. Hemolysis tests showed that SNS, at levels far exceeding the anticipated physiologic concentrations, did not induce red blood cell lysis. Further *in vitro* and *in vivo* experiments found that SNS did not activate platelets or cause coagulopathy and had no significant effects on the total number of blood cells or hepatorenal function. Cytotoxicity experiments suggested that SNS significantly inhibited the growth of tumor cells by damaging cell membranes, increasing intracellular ROS levels, inhibiting the release of TGF-*β*1 cytokines by macrophages, and inhibiting intracellular protein synthesis. In general, SNS appeared to have favorable biosafety and antitumor effects and may represent an attractive new therapeutic approach for the treatment of TNBC.

## 1. Introduction

Nanoparticle (NP) systems have arisen as a popular strategy among researchers seeking to improve methods of diagnosis and treatment for a wide range of diseases and have resulted in a number of approved diagnose/therapies such as Ferumoxytol, albumin-bound paclitaxel, and Onivyde [1–6]. Nanosystems are primarily used as drug loading or delivery carriers (e.g., for small molecules, proteins/peptides, or gene therapies) or directly as imaging agents [7–9]. Nanosystems as drug carriers for anticancer therapeutics have become a particularly popular field of research, given the potential benefits of targeted delivery to the tumor environment and a corresponding possibly reduction in systemic toxicities [10–12]. Nanomaterials have unique physical, chemical, and biological characteristics that allow them to effectively be loaded with chemotherapeutics or gene therapies [7–9]. Through active or passive transport that leads to preferential accumulation at the target site *in vivo*, the effective dose of chemotherapy drugs delivered to nontarget tissues can be significantly reduced, enabling either higher dosage to the tumor or reduced toxicity to the rest of the body [10, 11]. The PEGylated liposomal formulation of doxorubicin (Dox) reduces the toxicities and side effects of Dox through the enhanced permeability and retention effect (EPR) in the treatment of triple negative breast cancer (TNBC) [13, 14]. Similarly, nanocarrier-encapsulated indocyanine green (ICG) and Dox have allowed for combined treatment of hyperthermia and chemotherapy [15]. However, most of these new technologies are designed for systemic applications, raising biocompatibility as an important issue [16, 17].

In order to improve the poor prognosis of patients with TNBC that lacks specific, targetable receptors, our team designed a silica nanosystem (SNS) with a core made of Nano-Ag and an outer shell composed of mesoporous silica containing MnO_2_ blocked Dox, with anti-PD-L1 as a targeting group. The inclusion of anti-PD-L1 was aimed at capitalizing upon the highly expressed PD-L1 receptor on the surface of tumor cells and tumor-infiltrating lymphocytes (TIL) [18–20]. MnO_2_ itself has a role in chemodynamic therapy (CDT), and Dox is cytotoxic [21, 22]. Combined, this platform offers the potential for multimodal, synergistic treatment of TNBC. Prior preliminary studies showed that SNS can successfully target TNBC. In this work, to enable further potential clinical development of SNS, we investigated the biosafety and antitumor activity of SNS.

## 2. Materials and Methods

### 2.1. Materials

Assay kits for phorbol 12-myristate 13-acetate (PMA), reactive oxygen species (ROS), Triton X-100, BCA, IL-1*β*, and TGF-*β*1 were obtained from Beyotime Biotechnology (Shanghai, China). Kits for the measurement of adenosine diphosphate (ADP), lactate dehydrogenase (LDH), anti-PD-L1 (spartalizumab), and Dox were purchased from TOMUMS life science Co., Ltd. (Guangzhou, China). Cell Counting Kit-8 (CCK8) was obtained from Dojindo Laboratories (Shanghai) Co., Ltd. (Shanghai, China). Fetal bovine serum (FBS), penicillin, and streptomycin, Roswell Park Memorial Institute (RPMI) 1640 medium and Dulbecco modified Eagle medium (DMEM) were purchased from Thermo Fisher Scientific, Inc. (Waltham, USA). Other reagents not specifically listed here were obtained from Sinopharm (Beijing, China).

### 2.2. Cell Culture and Animal Experiments

Human Monocyte Leukemia Cell line THP-1, TNBC cell lines MDA-MB-231, and 4T1 were kindly provided by Stem Cell Bank of the Chinese Academy of Sciences. All cells were cultured at 37°C in 5% CO_2_, and the culture media for 4T1 and MDA-MB-231 cells was high-glucose DMEM with 1% penicillin-streptomycin and 10% FBS [2, 21]. The medium of THP-1 cells was RPMI 1640 medium with 1% penicillin-streptomycin and 10% FBS [16]. All animal studies met the Ethical Committee requirements of Bengbu College.

### 2.3. Preparation of SNS

(a) 7.5 mL glucose solution (30 mg/mL) was added to 40 mL of CTAB aqueous solution (1.25 mg/mL) and stirred at 80°C and 500 rpm for 30 min. Then, we slowly added 1.5 mL of a mixed solution containing 54 mg AgNO_3_ and 56 mg arginine. After 3 min, we added, in turn, 50 mg CTAB and 424 *μ*L TEOS and continued stirring for 3 h. Next, we added 15 mL of isopropanol, 30 mL of hydrochloric acid (5 N), and 20 mL of hexamethyldisilicyl ether (HMDO), which was then heated to 80°C and stirred for 30 min. The colored solution on the upper layer was collected, centrifuged, and washed many times with ethanol to obtain the silver-containing mesoporous silica NPs (MSN). (b) Next, we combined 25 mg of silver-loaded MSN and 1 mL of Dox·HCl (2.78 mg/mL and pH 7.0), which was stirred in the dark for 24 hours at room temperature to allow Dox adsorption to reach equilibrium. (c) The silver-loaded MSN and Dox were collected by centrifugation; then, we added 1 mL of KMnO_4_ solution (0.79 mg/mL) and 1.25 mL of MES (100 mm and pH 6.0) and mixed for 30 min by ultrasound. Then, the oxidation-reduction reaction between KMnO_4_ and MES formed brown MnO_2_, which would be loaded into the MSN-COOH shell to block Dox. Finally, MSN loaded with Nano-Ag, Dox, and MnO_2_ was obtained and precipitated by centrifugation and three washes (15,000 rpm and 15 min). (d) 20 mg MSN was dispersed in 5 mL MES (100 mm and pH 6), 1 mg anti-PD-L1 was added, and 0.2 mg EDC was added to crosslink for 4 h. Then, it was centrifuged and washed another 3 times (15,000 rpm and 15 min) and then freeze dried to obtain the final SNS.

### 2.4. Physicochemical Characterization

The diameter and size distribution of NPs were determined by dynamic light scattering (DLS) on a ZetaSizer Nano ZS (Malvern Instruments Ltd., UK). Zeta potential (*ζ*) was characterized using an He-Ne laser beam (*λ* = 633.8 nm) [23–26]. All measurements were made at 25°C, and the concentration of the sample in PBS solution was 40 *μ*g/mL. Ten microliters of NPs (40 *μ*g/mL) were dropped on a copper grid and dried at room temperature, then sputter-coated with gold and observed using an H-800 transmission electron microscope (TEM) (Hitachi, Tokyo, Japan) (acceleration voltage: 200 kV) [24–27]. The UV-Vis absorbance of different NP formulations was detected by GS54T UV-Vis spectrophotometer (Tianjin Tuopu Instrument Co., Ltd., Tianjin, China). All samples were analyzed in triplicate batches (*n* = 3).

### 2.5. Stability Evaluation of SNS

The size of SNS in PBS (pH 7.4), 2% BSA, and 5% FBS solution (NPs' final concentration: 40 *μ*g/mL) was detected using a ZetaSizer Nano ZS system after 6 h, 12 h, 24 h, 2 d, and 3 days of incubation at 37°C. We also measured the size of SNS in PBS of varying pH (5.0, 7.4, and 9.1) by DLS [25, 28].

### 2.6. Blood Compatibility

#### 2.6.1. Hemolysis Rate

Blood samples were collected from healthy male volunteers into an anticoagulant tube (BD EDTA routine blood tube) according to the anticoagulant tube instructions and diluted with normal saline (8 mL anticoagulant blood plus 10 mL normal saline). The prepared NPs were added to the diluted blood, and the final concentrations of NPs were 20, 50, 100, and 200 *μ*g/mL. After incubation for 1 h at 37°C, blood samples were centrifuged at 2,500 rpm for 5 min. The supernatant was collected, and the absorbance was measured using a UV-Vis spectrophotometer at 540 nm. Then, we calculated the hemolysis rate as the following formula (1):

(1) $$\begin{array}{c} \text{Hemolysis rate}\left(\%\right)=\left(\text{ODt}-\text{ODnc}\right)/\text{ODpc}\times 100\%, \end{array}$$

where ODt is the absorbance value of the NP group; ODnc is the absorbance value of the negative control (normal saline); ODpc is the absorbance value of the positive control (distilled water), and hemolysis rate was 100%. A hemolysis rate ≤ 5% indicated that the material met our safety requirements; a hemolysis rate > 5% indicated that the material could induce rupture of red blood cells (RBCs), thus failing requirements for clinical use [17, 23].

#### 2.6.2. Platelet Activation Analysis

To determine the platelet activation, fresh blood samples from healthy male volunteers were collected into sodium citrate anticoagulant tubes (BD Biosciences-China, China). Then, the blood was incubated with NPs at 37°C (final concentration: 200 *μ*g/mL). After 30 min, we assessed the degree of platelet activation by flow cytometry (FCM) (BD Biosciences, USA). For this, we measured the presence of fluorescently labeled platelet activation marker anti-CD62P and the platelet pan-marker anti-CD42a using FCM [11, 17]. 0.9% NaCl and 0.2 *μ*M ADP were used as negative control (NC) and positive control (PC), respectively.

#### 2.6.3. Effect of NPs on Blood Coagulation

Fresh blood samples were collected from healthy male volunteers into anticoagulation tubes with 109 mmol/L sodium citrate (*V*_blood_ : *V*_anticoagulant_ = 9 : 1). NPs were added to this blood sample to a final concentration of 200 *μ*g/mL. After incubation for 30 min at 37°C, the blood was centrifuged at 2,500 rpm for 10 min. Then, according to standard protocols, the activated partial thromboplastin time (APTT), prothrombin time (PT), thrombin time (TT), and fibrinogen (FIB) was measured on a Sysmex CS5100 Automatic Coagulation Analyzer (Xisen Meikang Medical Electronics (Shanghai) Co., Ltd., Shanghai, China) [29]. PBS and hemocoagulase atrox (0.1 unit/mL) were used as NC and PC, respectively.

#### 2.6.4. Effect of NPs *In Vivo* on Blood Cells and Biochemical Parameters

NPs (1.5 mg/kg) were injected into the tail vein of female SD mice (7-8 weeks) once every 7 d. We used PBS and Dox (4.5 mg/kg) as the NC and PC. After 14 d, the mice were sacrificed, and fresh blood was collected into an anticoagulant tube with 109 mmol/L sodium citrate (*V*_blood_ : *V*_anticoagulant_ = 9 : 1). A complete blood count and clinical blood chemistries (to assess liver and kidney functions) were measured using a Sysmex pocH-100i Automated Hematology Analyzer (Sysmex Medical Electronics (Shanghai) Co., Ltd., China) and an AU480 Beckman Automatic Biochemical Analyzer (Beckman Coulter, Inc., Miami, USA), as instructed by the manufacturer [16].

### 2.7. Cell Compatibility

#### 2.7.1. LDH Release Experiment

The integrity of cell membranes was determined by an LDH release experiment. An appropriate amount of 4T1 and MDA-MB-231 cells were cultured in 96-well plates, per kit instructions. After adherence, the culture medium was aspirated and 200 *μ*L of DMEM containing NPs (5 *μ*g/mL) was added into each well of the 96-well plates. DMEM medium containing 1% Triton X-100 was used as the PC, and DMEM medium alone was used as the NC [2, 16]. After 24 h, the LDH concentration in the supernatant was determined according to the LDH kit instructions.

#### 2.7.2. Intracellular ROS Level Assay

Intracellular ROS levels were determined using 2,7-dichlorodihydrofluorescein diacetate (DCFH-DA) as the indicator. In brief, an appropriate amount of 4T1 and MDA-MB-231 cells were incubated in 6-well plates. After adherence, DMEM medium containing NPs (5 *μ*g/mL) was added and cells were cultured. DMEM medium containing H_2_O_2_ (50 *μ*M) was used as the PC. PBS was used as the NC [16, 30]. After 6 h, the cells were washed 3 times using PBS. Then, the effect of NPs on intracellular ROS levels was determined per the kit's instructions.

### 2.8. Immunocompatibility

PMA was added into RPMI 1640 medium containing THP-1 cells in the logarithmic phase of growth to a final concentration of 50 ng/mL. Then, the cell suspension medium was added to 96-well plates at 200 *μ*L per well and incubated for 48 h to induce THP-1 cells to differentiate into macrophages. Then, the RPMI 1640 was carefully aspirated and replaced by 200 *μ*L of RPMI 1640 medium containing NPs (concentration: 5 *μ*g/mL). In this experiment, 200 *μ*L RPMI 1640 medium was used as the NC, and 200 *μ*L RPMI 1640 medium containing 2.5 mg/mL of inulin was used as the PC. Cells were cultured for another 24 h, and the supernatant was measured for absorbance at 450 nm according to the IL-1*β* and TGF-*β*1 quantitative enzyme-linked assay kit instructions [16]. We used a standard curve to determine the contents of IL-1*β* and TGF-*β*1 in the medium.

### 2.9. Protein Synthesis Experiment

4T1 cells and MDA-MB-231 cells were cultured in 6-well plates. After adherence, the culture medium in the 6-well plates was aspirated, and 1,000 *μ*L DMEM containing NPs (concentration: 5 *μ*g/mL) was added to each well. PBS and free Dox (10 *μ*g/mL) were used as the NC and PC, respectively. After incubation for 24 h, protein concentration was measured according to the instructions of the BCA kit to evaluate the effects of different NPs on protein synthesis and cytotoxicity [31, 32].

### 2.10. Statistical Analysis

Data were presented as mean ± standard deviation (SD). One-way ANOVA and an unpaired Student's *t*-test were used to determine the statistical significance of cross-group comparisons. A threshold of *p* < 0.05 was considered statistically significant.

## 3. Results

### 3.1. Physicochemical Characterization

**Figure 1:**
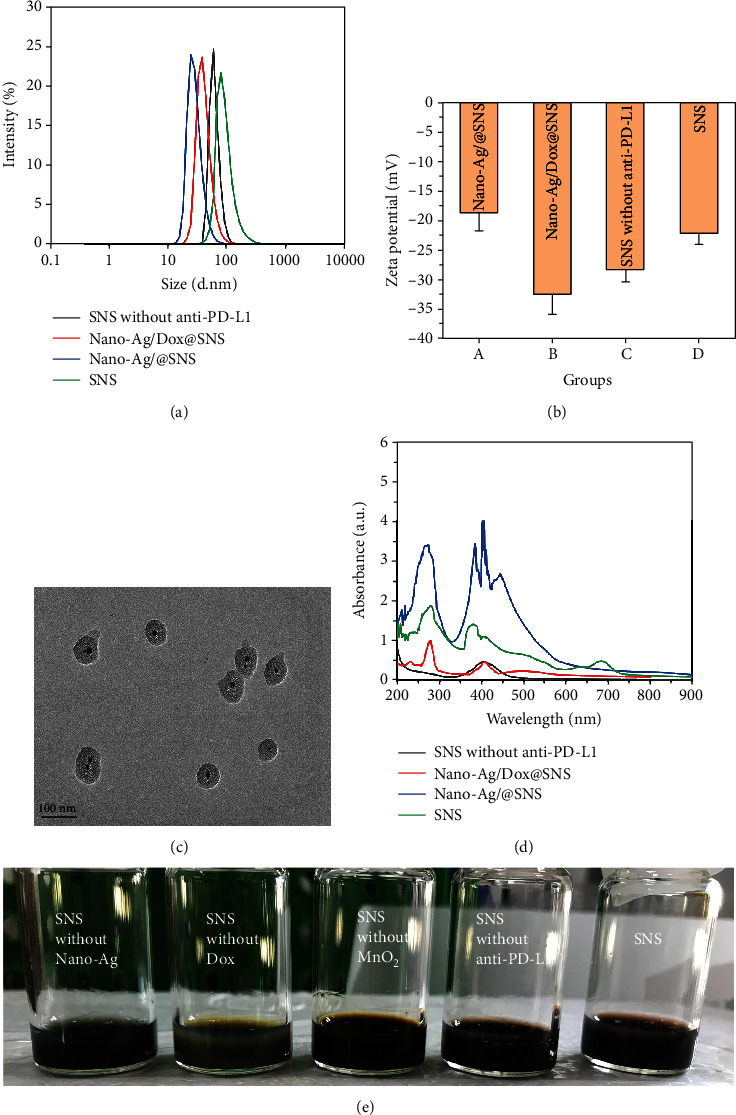
Characterization of different NPs during SNS preparation. (a) The particle size and (b) zeta potential of NPs at different stages of SNS preparation; (c) TEM of SNS showed that SNSs were uniform spheres with no aggregation; (d) UV-Vis absorption spectra of NPs in different stages of SNS preparation; (e) the appearance of different NPs in distilled water.

During the preparation of SNS, the particle size of the intermediate NPs was about 50–80 nm (Figure 1(a)). After loading MnO_2_, the size of NPs obviously increased, indicating that MnO_2_ played a role of sealing mesoporous on the surface of mesoporous silica. After NPs were conjugated with anti-PD-L1, the hydrodynamic diameter also slightly increased (by about 94.6 nm). The Dox loaded into NPs was fully encapsulated after the addition of MnO_2_, and the zeta potential of the intermediate NPs was between -20 and -30 mV. The zeta potential of full SNS was -22.1 mV (Figure 1(b)). TEM images of SNS showed that the size distribution of the black core Nano-Ag was uniform, and the morphology of the SNS with 90-100 nm diameter was uniformly spherical, in good agreement with the DLS findings of 94.6 nm (Figure 1(c)). In the UV-Vis absorption spectra, SNS without anti-PD-L1 and SNS evinced high signal peaks are representing Nano-Ag (408 nm), Dox (272 nm), and MnO_2_ (380 nm) (Figure 1(d)). The signal peaks of Dox/Ag@SNS had Nano-Ag (408 nm) and Dox (272 nm). As expected, Ag@SNS only had peaks for Nano-Ag (408 nm). These findings indicated that the Nano-Ag, Dox, and MnO_2_ were successfully coloaded into SNS prior to anti-PD-L1 grafting. After SNS preparation, the content of Dox in the supernatant was detected by a UV-Vis spectrophotometer, and the drug-loading rate of Dox was determined to be about 10.2%. As seen in Figure 1(e), the SNS without Nano-Ag was dark blue, and the SNS without Dox was yellow green. SNS, SNS without MnO_2_, and SNS without anti-PD-L1 were color of MnO_2_: light brown.

### 3.2. Stability of SNS

**Figure 2:**
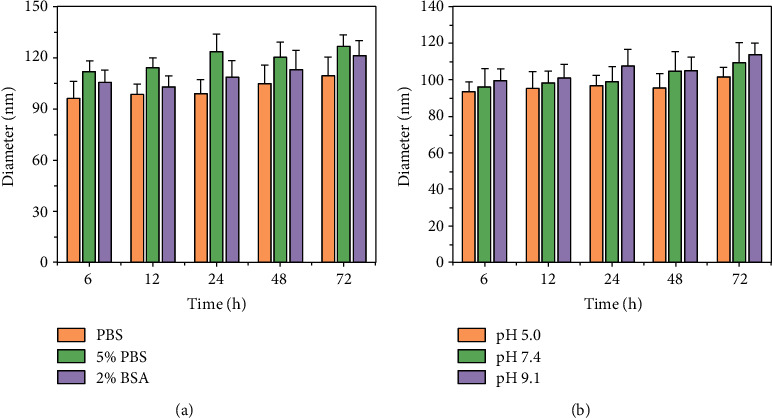
The stability of SNS in various buffers was determined by DLS. The change in size of SNS in (a) different media and (b) PBS of varying pH, over various incubation times. The size of SNS did not significantly change relative to preincubation SNS.

When NPs are injected to systemic circulation, they adsorb plasma proteins that change the size of NPs and extent of phagocytosis by macrophages, thus affecting *in vivo* distribution and retention time [33, 34]. Before SNS incubation with PBS, 2% BSA, and 5% FBS, the particle size was 94.6 nm by DLS. In a PBS alone, PBS with 5% FBS, and PBS with 2% BSA, the particle sizes of SNS at 12 h were 98.52 nm, 114.11, nm and 103.04 nm, respectively; at 72 h, the average diameters were 109.39 nm, 126.92 nm, and 121.41 nm, respectively (Figure 2(a)). Thus, protein contents in buffer appeared to slightly increase mean particle size, potentially due to protein adsorption. However, these differences were not statistically significant. With prolonged incubation time, the particle size of SNS in different pH values of PBS solution gradually increased (Figure 2(b)). At 12 h, in PBS solutions with pH of 5.0, 7.4, and 9.1, the size of SNS was 95.16 nm, 98.52 nm, and 101.09 nm, respectively; at 72 h, the sizes were 101.73 nm, 109.39 nm, and 113.74 nm, respectively. The lower the pH value, the smaller the increase in SNS particle size; the larger the pH value, the larger the increase in SNS particle size. However, compared with the preincubation of SNS diameters, there was no statistically significant difference, potentially due to the accelerated degradation of MnO_2_ in acidic solution [31]. These results suggest that SNS is stable over a range of physiologic buffer conditions.

### 3.3. Blood Compatibility

#### 3.3.1. Hemolysis Rate

**Figure 3:**
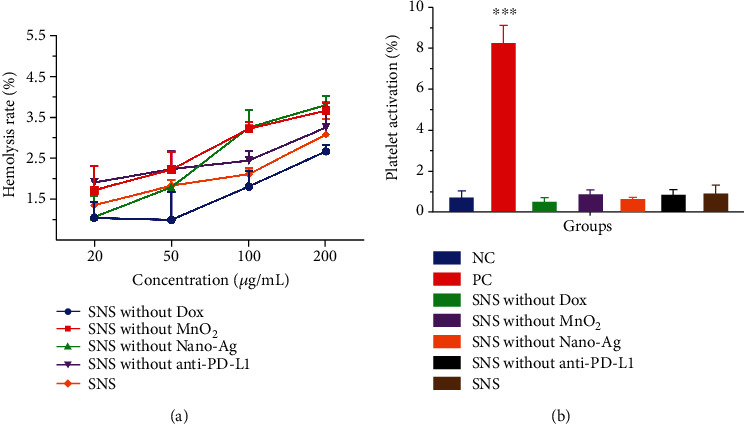
Blood compatibility of NPs. (a) The hemolysis rate of NPs at different concentrations and different stages of preparation. The hemolysis rate of all NPs at 200 *μ*g/mL was less than 4%, suggesting that SNS had no significant effect on RBC rupture. (b) The effect of different forms of NPs on platelet activation. Treatment with 200 *μ*g/mL NPs led to no significant induction of platelet activation, and there was no significant difference compared with the NC (0.9% NaCl), but the NPs and NC were far lower than the PC (0.2 *μ*M ADP). ^∗∗∗^ indicates that, compared with NC, *p* < 0.001.

*In vitro* hemolysis tests are considered an important and reliable method to evaluate the hemocompatibility of drugs [35]. In these hemolysis experiments, we assessed for a linear relationship between SNS concentration and hemolysis rate. The fitted linear equation of SNS without Dox was *y* = 0.7612 + 0.00966x (*R*^2^ = 0.96); for SNS without MnO_2_ group was *y* = 1.7471 + 0.01053x (*R*^2^ = 0.88); SNS without Nano-Ag group was *y* = 1.1693 + 0.01460x (*R*^2^ = 0.87); SNS without anti-PD-L1 group was *y* = 1.8149 + 0.007144x (*R*^2^ = 0.99); and, finally, SNS group was *y* = 1.2731 + 0.009075x (*R*^2^ = 0.99). When the above NPs reached 5% hemolysis rate, the concentrations were 438.80, 308.92, 262.38, 445.84, and 410.68 *μ*g/mL, respectively, significantly higher than the maximum experimental concentration of 200 *μ*g/mL used in this experiment. Moreover, when the maximum experimental concentration of NPs was 200 *μ*g/mL, the hemolysis rate of all NPs was less than 4% (Figure 3(a)). These experiments suggest that the extent of RBC rupture induced by SNS over the relevant concentration range (5-10 *μ*g/mL) is likely to be far less than 5%. According to the standard of American Society for Materials Testing (ASTM F756-00, 2000), SNS and other NPs are not typically hemolytic [25].

#### 3.3.2. Platelet Activation Test

Platelet activation is a complicated process involving many physiological and pathological processes, including thrombosis events, inflammation, tumor growth, and metastasis [11, 17]. When foreign NPs are introduced to the blood, the degree of platelet activation is a key indicator of hemocompatibility. We performed a platelet activation test to study the hemocompatibility of SNS, by FCM (Figure 3(b)). The platelet activation results suggested that NPs were not significantly different than NC, which had very low platelet activation (*p* > 0.05). The PC, however, induced clear platelet activation, and there was a significant difference between PC and NC (*p* < 0.001). These results indicated that SNS likely has good platelet compatibility.

#### 3.3.3. Effects of SNS on Coagulation

PT is the coagulation status screening test for extrinsic coagulation system and assesses for the function of each coagulation factor. The PT is an important index for monitoring patients on anticoagulation treatment. When the test value is more than 3 s greater than the control value, it is considered abnormal [16]. Similarly, APTT is a test that reflects the coagulation status of the intrinsic coagulation system and is often used as a screening test [25]. TT is a simple test to detect the function of the coagulation, anticoagulation, and fibrinolysis systems [25]. In addition, when a platelet is activated, it will release a coagulase activator, which catalyzes prothrombin to turn into thrombin in a manner dependent on calcium ions. Thrombin converts water-soluble FIB in the plasma into water-insoluble fibrin. FIB accumulates around blood cells, binding them together to form a mass which results in a clot [16]. The normal concentration of FIB (cFIB) is 2-4 g/L. In the clinic, PT, APTT, TT, and cFIB are generally used as convenient indices for coagulation screening. In our study, when comparing NC and different NP groups, there was no significant difference in results from PT, APTT, TT, and cFIB (Table 1). Comparing the PC and NC, the PT and TT were not significantly different, but the APTT and cFIB in the PC were significantly lower than NC (*p* < 0.05). Together, these results suggest that SNS did not obviously affect the coagulation system. These results are also in accordance with the mechanism of action of hemocoagulase atrox, which contains two enzymes that coagulate blood: thrombin-like and thrombokinase-like. The former promotes platelet aggregation at the site of bleeding to form white thrombus (platelet thrombus) and produce hemostasis. The latter is activated by platelet factor III (phospholipid), which converts prothrombin into thrombin and further activates fibrinogen to become fibrin [36]. Therefore, it is fitting that hemocoagulase atrox would primarily affect APTT and cFIB.

#### 3.3.4. Effect of NPs on Blood Component Results

The effects of treatment on blood cells and biochemical parameters are listed in Table 2. Between the NC and NP groups, there was no significant difference in white blood cells (WBC), hemoglobin (HB), platelet (PLT), alanine aminotransferase (ALT), aspartate aminotransferase (AST), serum creatinine (Scr), and blood urea nitrogen (BUN) over the course of treatment. In the PC, compared with NC, the biochemical parameters (ALT, AST, BUN, and Scr) were significantly increased (*p* < 0.05), and the blood cell counts (WBC, HB, and PLT) were significantly decreased (*p* < 0.05). These results indicated that the SNS did not significantly affect blood cells or biochemical parameters, suggesting that SNS is not likely to be toxic *in vivo.*

### 3.4. Cell Compatibility

#### 3.4.1. LDH Release Experiment

**Figure 4:**
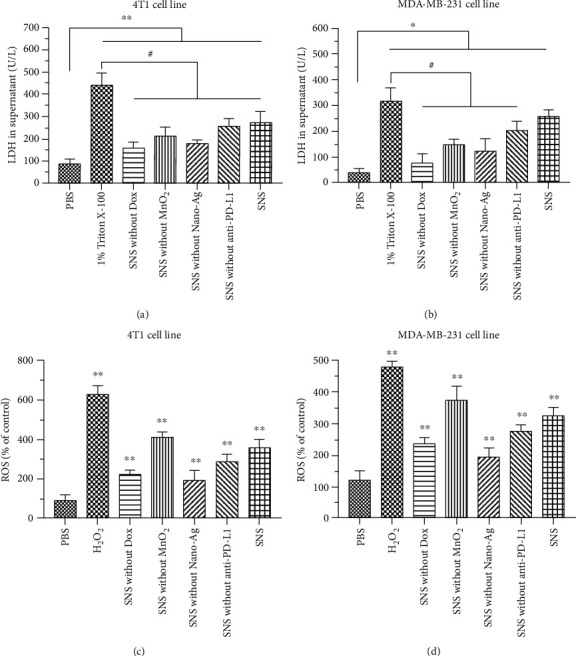
LDH release and intracellular ROS assays. The extent to which NPs induced LDH release in (a) 4T1 cells and (b) MDA-MB-231 cells. The extent to which NPs induced intracellular ROS in (c) 4T1 cells and (d) MDA-MB-231 cells. ^∗^ and ^∗∗^ indicate that, compared with NC, *p* < 0.05 and *p* < 0.01, respectively. ^#^ indicates that *p* < 0.05 relative to PC.

LDH is an endoenzyme that cannot pass through the intact cell membrane. Therefore, LDH release experiments can be used to determine whether treatment with SNS leads to cell membrane damage. The LDH concentration in medium at 24 h is shown in Figure 4(a) and Figure 4(b). The LDH concentrations of the NC in the 4T1 cells (87.56 U/mL) were significantly lower than the PC (442.52 U/mL) (*p* < 0.01). The LDH concentrations of SNS without Dox, SNS without MnO_2_, SNS without Nano-Ag, SNS without anti-PD-L1, and SNS in 4T1 cells were 159.66, 213.04, 178.23, 256.81, and 274.09 U/mL, respectively. The LDH concentration of these NP groups was significantly higher than NC (*p* < 0.01) and significantly lower than PC (*p* < 0.05). Moreover, SNS was associated with the highest extent of LDH release. We observed similar behavior in the MDA-MB-231 cells, except that the LDH concentration of the SNS group was not significantly different compared to the PC. The high LDH release after treatment with NPs was indicative of NP-induced damage to the cell membrane. These results suggest that SNS alone leads to the most significant damage to the cell membrane.

#### 3.4.2. Intracellular ROS Assay

Excessive ROS in cells can destroy proteins, DNA, phospholipids, and other biological macromolecules. The generation of ROS is one main anticancer mechanism of radiotherapy and several therapeutic agents [1, 37–39]. Therefore, it is of great clinical and theoretical significance to measure the effect of NPs on intracellular ROS production. DCFH-DA has no fluorescence and can freely pass through the cell membrane. After entering the cell, DCFH-DA in the cells can be hydrolyzed by esterase to generate DCFH. Importantly, DCFH cannot penetrate the cell membrane, so the DCFH-DA probe remains inside the cell. Intracellular ROS oxidizes the nonfluorescent DCFH to produce fluorescent DCF [1, 31]. Thus, the intracellular ROS level can be measured by detecting the fluorescence of intracellular DCF. The intracellular ROS levels are shown in Figure 4(c) and Figure 4(d). The intracellular ROS of 4T1 cells treated with NC (91.39%) was significantly lower than that of PC (628.65%) (*p* < 0.01). The intracellular ROS level produced after treatment with SNS without Dox, SNS without MnO_2_, SNS without Nano-Ag, SNS without anti-PD-L1, and SNS in 4T1 cells was 225.89%, 413.54%, 194.33%, 289.37%, and 357.09%, respectively. The intracellular ROS levels after treatment with each of these NP groups were significantly higher than NC (*p* < 0.01) but lower than PC (*p* < 0.01). SNS without MnO_2_ led to the highest intracellular ROS level among all SNS variants, perhaps due to the ability of MnO_2_ to react directly with ROS. We found similar trends in the MDA-MB-231 cells. These results indicate that SNS may have antitumor activity by producing ROS.

### 3.5. Immunocompatibility

The polypeptide IL-1*β* is mainly produced by monocytes and macrophages and contributes to inflammation that can result in fever. However, in the tumor microenvironment, it can cause tumor growth and metastasis [2, 39]. TGF-*β*1 is mainly expressed by endothelial cells, blood cells, connective tissue cells, and epithelial cells. TGF-*β*1 blocks the differentiation of immature T cells into Th1 cells, promotes their transformation into Treg subsets, and inhibits the antigen-presenting function of dendritic cells, thus interfering with normal immune regulation and potentiating immune escape of tumor cells [24, 40]. Therefore, it is of great significance to evaluate the effects of SNS on the release of IL-1*β* and TGF-*β*1 by monocytes and macrophages.

**Figure 5:**
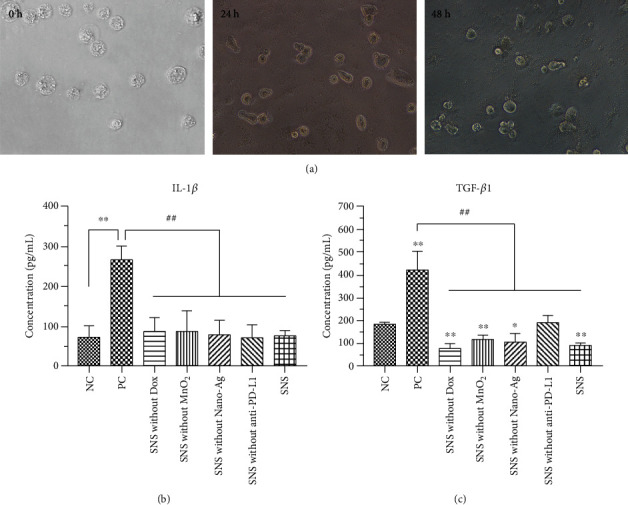
The effects of NPs on cytokine release. (a) THP-1 cells were induced and differentiated into macrophages by treatment with PMA for 0, 24, and 48 h. (b) Macrophages were incubated with the different NPs for 24 h, and the concentrations of (b) IL-1*β* and (c) TGF-*β*1 released from cells were significantly lower than that of PC. For IL-1*β*, there was no significant difference between the NC and NP groups. For TGF-1*β*, there was a significant difference between NC and SNS (*p* < 0.01). ^∗^ and ^∗∗^ indicate *p* < 0.05 and *p* < 0.01, respectively, compared with NC. ^##^ indicates *p* < 0.01 compared with PC.

THP-1 monocytes were induced to differentiate by treatment with PMA for 48 h, and we observed fusiform, elliptical, or irregular adherent cells (Figure 5(a)). This observation suggests that THP-1 cells were successfully differentiated into macrophages. In the following immunocompatibility test, the medium IL-1*β* concentrations after treatment with NC (72.08 ± 27.44 pg/mL) were significantly lower than PC (266.98 ± 32.47 pg/mL) (*p* < 0.01) (Figure 5(b)). The IL-1*β* level after treatment with SNS without Dox, SNS without MnO_2_, SNS without Nano-Ag, SNS without anti-PD-L1, and SNS was 87.03 ± 33.61, 87.93 ± 49.95, 78.16 ± 36.02, 70.93 ± 31.64, and 76.17 ± 11.51 pg/mL, respectively. The IL-1*β* concentrations of these NP groups had similar concentrations as the NC (*p* > 0.05), but significantly lower than the PC (*p* < 0.01). These results suggested that treatment of macrophages with SNS did lead to the synthesis and release of IL-1*β*. The TGF-*β*1 concentrations after incubation with NC (183.24 ± 7.90 pg/mL) were significantly lower than after treatment with PC (421.59 ± 79.61 pg/mL) (*p* < 0.01) (Figure 5(c)). The TGF-*β*1 concentrations after treatment with SNS without Dox, SNS without MnO_2_, SNS without Nano-Ag, SNS without anti-PD-L1, and SNS were 79.13 ± 19.14, 118.01 ± 16.74, 92.67 ± 40.08, 164.80 ± 66.10, and 91.34 ± 8.91 pg/mL, respectively. The TGF-*β*1 concentrations of these NP groups (except SNS without anti-PD-L1) were significantly lower than NC (*p* < 0.05) and PC (*p* < 0.01). The TGF-*β*1 concentrations resulting from treatment with SNS without anti-PD-L1, however, was slightly higher than after treatment with the NC (*p* > 0.05). These indicated that targeting with anti-PD-L1 leads to decreased release of TGF-*β*1, potentially further enhancing the immunotherapy benefits of SNS. However, anti-PD-L1 in SNS did not affect the release of IL-1*β*.

### 3.6. Intracellular Protein Synthesis

**Figure 6:**
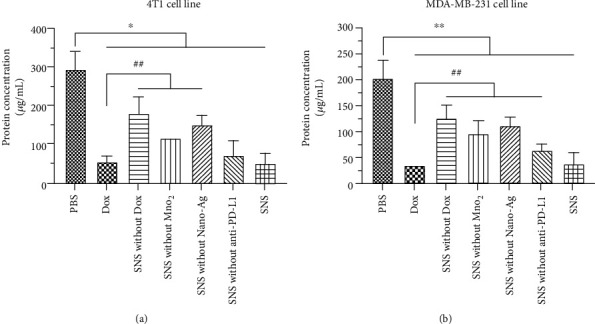
After (a) 4T1 cells and (b) MDA-MB-231 cells were treated with different NPs (5 *μ*g/mL), the effects of NPs for intracellular protein synthesis were evaluated. ^∗^ and ^∗∗^ indicate that intracellular protein concentrations were significantly lower than that of cells treated with NC, at *p* < 0.05 and *p* < 0.01, respectively. ^##^ indicates that the intracellular protein concentration was significantly higher than PC, *p* < 0.01. The intracellular protein content of the SNS-treated group was the lowest, and SNS had the strongest antitumor activity among all NP groups.

The intracellular protein concentrations are shown in Figure 6. 4T1 cells had significantly different intracellular protein after treatment with NC, relative to after treatment with PC or NPs (*p* < 0.05). The intracellular protein levels after treatment with SNS without Dox, SNS without MnO_2_, or SNS without Nano-Ag were significantly higher than PC (*p* < 0.01). There was no significant difference between PC with SNS and SNS without anti-PD-L1 (*p* > 0.05). These findings indicate that SNS without anti-PD-L1 and SNS groups have a comparable antitumor activity as the PC. Moreover, the cytotoxicity of SNS (as indicated by impact on intracellular protein concentration) was the highest in all SNS variants. In the MDA-MB-231 cells, the intracellular protein concentration after treatment with SNS without Dox, SNS without MnO_2_, SNS without Nano-Ag, and SNS without anti-PD-L1 was significantly higher than after treatment with PC (*p* < 0.01). The cells treated with SNS had similar intracellular protein concentrations as cells treated with PC (*p* > 0.05). The intracellular protein concentration of all NP-treated cells was lower than that of the NC (*p* < 0.01). These results indicated that all NPs had some level of cytotoxicity, but the final formulation of SNS (with targeting ligand) had the strongest antitumor activity. This may be due to the targeting and immunotherapy activity of anti-PD-L1. This result further confirmed the data obtained in the LDH release.

## 4. Discussion

In recent years, immunotherapies and targeted therapies for the treatment of malignant tumors have been an area of rapid scientific and clinical development [6, 18]. However, these approaches have not yet been translated to meaningful improvements in outcomes for patients with TNBC [41, 42]. Therefore, in this work, we sought to test a new nanodelivery system to potentially leverage anti-PD-L1 activity to achieve targeted immunotherapy. Prior indications were supporting the potential effectiveness of this approach, so we required a better understanding of the biocompatibility of SNS.

In this study, we used DLS to analyze the stability of SNS, as indicated by changes in particle size. Upon injection, plasma proteins are adsorbed to NPs, forming a hydrodynamic corona on the NPs' surface and thus affecting the particle size and zeta potential of NPs, ultimately changing the retention time of NPs by altering interactions with the reticuloendothelial system (RES) and renal filtration [43, 44]. Generally, smaller particles are less likely to be removed by the RES, leading to a more favorable pharmacokinetic and biodistribution profile. Additionally, smaller particles also have a more hydrophilic surface, potentially decreasing protein adsorption [34]. However, if particles are too small, such as below 10 nm, they are more likely to extravasate from the liver and kidney. It has been reported that NPs with a particle size of about 100 nm are most efficient at avoiding scavenging by the RES and benefiting from the EPR effect to distribute into tumor tissue [44, 45]. We found that when the incubation time was prolonged, the size of about 100 nm SNS did not change regardless of incubation in PBS, 5% FBS, and 2% BSA suggesting good stability. The average particle size of SNS incubated in 2% BSA was slightly smaller than those incubated in 5% FBS, potentially because BSA is an extracted albumin (583 amino acid residues, molecular weight 66.43 kDa) from bovine serum, and its molecular weight is smaller than that of the full proteins found in FBS. We found that incubation in buffers with lower pH led to smaller SNS, potentially because MnO_2_ decomposed more quickly and could improve the state of cell hypoxia. This phenomenon may help improve the antitumor effects of SNS in acidic organelles of tumor cells.

Since it would likely be administered via infusion to the systemic circulation, understanding the hemocompatibility of SNS is crucial to understand its safety profile *in vivo*. According to the international standard (ISO 10933-4), the blood compatibility of synthetic materials is mainly considered in the following two respects: (1) whether the therapeutic does not damage blood components (such as by changing hemolysis rate, hepatorenal function, or blood cell counts); (2) whether the therapeutic does not cause thrombosis (i.e., by induction platelet activation and coagulation) [16, 46].

In this paper, the total blood hemoglobin (TBH) and plasma-free hemoglobin (PFH) released into the plasma were determined by quantitative colorimetry [47]. For these toxicity studies, we tested concentrations of NPs in vast excess of what we might expect to use during treatment. Generally, the experimental concentration of NPs should be at least 30 times of the expected treatment concentration. The expected therapeutic concentration of SNS is roughly 5 *μ*g/mL, so we tested at a concentration of 200 *μ*g/mL.

In our study, the hemolysis rate reached 5% when the concentration of NPs was 262.38 *μ*g/mL according to the linear regression. Therefore, SNS at therapeutic concentrations (5 *μ*g/mL) likely has no significant effect on hemolysis. This was an expected result, because RBC damage typically would result from electrostatic adsorption and hydrophobic interactions. However, in this study, the hydrophobic mesoporous silica surface contains hydrophilic carboxyl groups and can be really dissolved in water. Although SNS has a negative charge, the hydrophobic interactions between SNS and RBCs are greater than the electrostatic repulsions between SNS and RBCs. Therefore, at high concentrations, SNS can induce the rupture of RBC membranes.

During chemotherapy for malignant tumors, myelosuppression is a common serious side effect. This is because chemotherapy leads to a decrease in bone marrow hematopoietic capacity, decreasing the number of peripheral blood cells. Second, due to the varying half-lives of various blood cells, the initial manifestation of myelosuppression is usually leukopenia, especially neutropenia, followed by PLT and a reduction in hemoglobin [48]. Therefore, it is of great significance to monitor changes in RBCs, white blood cells, and platelets after the administration of cytotoxic drugs.

ALT is mainly present in hepatocytes (but not in their mitochondria). On the other hand, AST is mainly distributed in the mitochondria of cells in the heart and liver. The plasma concentrations of these two enzymes are very low under healthy conditions. However, when hepatocytes are damaged, the permeability of cell membranes increases, leading to the release of these two enzymes from the liver cells. The kidney is primarily responsible for blood filtration and waste removal, so it plays a key role in the transport and removal of NPs. When NPs enter the body and are phagocytosed by the RES, NPs are primarily concentrated in the liver, spleen, and kidney, potentially affecting the liver and kidney function and resulting in abnormal increase of ALT, AST, Scr, and BUN [16]. We found that after 14 days of PC treatment in mice, blood cell counts were significantly reduced and biochemical parameters were significantly increased. However, treatment with SNS or NC did not lead to significant differences in blood cells or biochemical parameters, despite loading with Dox and Ag. This is likely because of the protective effects of drug encapsulation by the mesoporous silica carrier, which prevented direct contact between Dox, Ag, and normal tissue.

Platelet activation can result from three main mechanisms. (1) ADP pathway: ADP, thrombin, adrenaline, etc. induce platelets to release endogenous ADP and cause platelet aggregation; (2) TXA2 pathway: PGG2 and TXA2 induce platelet aggregation; (3) PAF pathway. SNS, as an exogenous substance entering the blood, may induce platelet activation and aggregation through any of the above pathways to cause thrombosis. In addition, NPs with negative charges have been shown to induce platelet aggregation more strongly than cationic or neutral NPs [2, 11]. The zeta potential of SNS is negative, so it was important to evaluate the platelet aggregation effects of SNS. In our study, SNS and 0.9% NaCl solution did not induce platelet aggregation, but PC did, indicating that SNS had no significant effect on the platelet coagulation pathway.

Next, we further studied the effects of SNS on coagulation. Blood vessel damage or the introduction of exogenous substances can cause activation of the coagulation cascade, leading to the generation of thrombin, and the conversion of fibrinogen into fibrin, which promotes coagulation [49]. This can be divided into three basic steps: the formation of the prothrombin complex, the activation of prothrombin, and the formation of fibrin. Coagulation function is mainly divided into the endogenous coagulation system, exogenous coagulation system, and fibrinolysis system. In the clinic, APTT, PT, TT, and cFIB are common tests used to evaluate coagulation function [25, 49]. In our study, the APTT and cFIB of hemocoagulase atrox (our PC) were significantly higher than that of the NPs and NC, suggesting that the coagulation assay was functioning correctly. However, the coagulation function was not significantly different between the NP group and NC group, suggesting that SNS had no significant effect on coagulation function. This could be due to the hydrophilicity of the SNS shell, which may reduce the phagocytosis of NPs. Further, SNS has the same charge as many plasma proteins, potentially decreasing opsonization.

LDH is a NAD-dependent kinase. It can be divided into NAD-dependent-L-lactate dehydrogenase and NAD-dependent-D-lactate dehydrogenase. LDH mainly exists in the cytoplasm and cannot penetrate the cell membrane under normal conditions. When the cell membrane is damaged, however, LDH can exit the cell [16, 50]. Thus, LDH activity in culture medium is reflective of the cytotoxicity of NPs. In our study, SNS resulted in the largest amount of LDH release among all NP groups and was significantly higher than NC. These results are consistent with our finding that SNS also led to the greatest decrease in intracellular protein concentration. Thus, the observed increase of LDH release is likely due to cell death, not just cell membrane damage.

ROS consists of free and nonfree radicals from oxygen sources, including superoxide anion (O^2-^), H_2_O_2_, hydroxyl radical (OH-), ozone (O_3_), and singlet oxygen (1O_2_). These each possess unpaired electrons, resulting in high chemical reactivity. In the natural biological setting, ROS is a by-product of oxygen metabolism and plays an important role in cell signaling and homeostasis [51, 52]. However, ROS levels can dramatically increase during environmental pressures (e.g., after UV or heat exposure), potentially causing oxidative stress or serious damage to cell structures [1, 31]. Thus, we can measure possible cytotoxicity by determining the effect of SNS on ROS production and metabolism. Among all tested NPs, SNS without MnO_2_ led to the highest level of intracellular ROS, likely because the MnO_2_ reacts with H_2_O_2_ in acid environments to produce O_2_, increasing ROS consumption and leading to a state of oxygen deficiency [31]. SNS without Nano-Ag resulted in the lowest level of intracellular ROS, potentially because Nano-Ag can also generate ROS. SNS has MnO_2_ and Nano-Ag, so the ROS resulting from treatment with SNS was at a level between that induced by SNS without MnO_2_ and SNS without Nano-Ag. The ROS resulting from treatment with SNS without anti-PD-L1 was lower than that of SNS, further supporting the cytotoxic effects of the anti-PD-L1 targeting moiety, in good agreement with results from the LDH release test and intracellular protein assays.

IL-1*β* family cytokines play an important role in host defense mechanisms as well as the pathogenesis of various immune diseases. Local overexpression of IL-1*β* in the early chronic inflammatory environment promotes tumor development. After tumorigenesis, IL-1*β* interacts with vascular endothelial growth factor (VEGF) in the tumor microenvironment to drive angiogenesis and enable tumor metastasis and diffusion [53, 54]. In addition, IL-1*β* also supports metastasis by promoting the transformation of cancer stem cells and the epithelial mesenchymal transition (EMT). In the tumor microenvironment, tumor cells also produce IL-1*β* and act on other cells through autocrine function, thereby avoiding apoptosis and promoting proliferation and invasion [55]. At the tumor site, low-level IL-1*β* expression usually induces immunosuppression at the early stage of disease, but high-level IL-1*β* usually leads to cell invasion [40].

As a growth factor, TGF-*β*1 exerts biological functions regulating cell proliferation, differentiation, apoptosis, and immunity. TGF-*β*1 induces tumor cell apoptosis and inhibits tumor growth by regulating the downstream signal transduction molecule Smad in the early stages of tumor development [55, 56]. With further development of the tumor, gene mutations in the TGF-*β*1 receptor or its downstream Smad pathway accumulate in tumor cells, resulting in the weakening of TGF-*β*1 inhibition [55]. The activation of the TGF-*β*1 receptor promotes EMT, and polarized epithelial cells can be transformed into active stromal capable of invasion and migration. This process is a crucial stage of tumor occurrence, growth, and metastasis [56, 57]. TGF-*β*1 is a chemokine that can attract macrophages and fibroblasts and cause the release of bFGF, PDGF, TNF-a, and other vasoactive factors, thus promoting angiogenesis and inducing tumor metastasis [24, 56]. In this study, THP-1 cells were successfully induced and differentiated into adherent macrophages, and the IL-1*β* release of the SNS-treated group was not significantly different from NC groups. However, the extent of TGF-*β*1 release in the SNS-treated group was significantly lower than that of the NC groups. The TGF-*β*1 had the capability to promote tumor development. These findings indicated that SNS can enhance the antitumor effects by TGF-*β*1 pathway. The TGF-*β*1 release due to treatment with SNS without anti-PD-L1 was significantly greater than the SNS-treated groups. This finding supports the importance of the anti-PD-L1 targeting moiety in the immunotherapeutic effects of SNS.

Assays of intracellular protein synthesis are interpreted similar to the results of the MTT assay and are a reflection of the cytotoxicity of drugs. The results of intracellular protein and LDH release assays were consistent, suggesting that treatment with SNS led to strong cytotoxicity and may represent a promising strategy for targeted immunotherapy.

## 5. Conclusions

There remains a strong need for improved therapies of difficult-to-target cancers such as TNBC. In this work, we assessed the cytotoxicity and biocompatibility of SNS, a new candidate therapeutic platform. The results of TEM and DLS suggested spherical particles of about 95 nm with no tendency for aggregation. UV-Vis spectrum analysis of SNS suggested that SNS was successfully loaded with Nano-Ag, Dox, and MnO_2_, and the drug-loading rate of Dox was about 10.2%. Stability tests showed that the particle size of SNS in PBS, 5% FBS, and 2% BSA solution did not significantly change within 72 hours. Meanwhile, we found that incubation in solutions of lower pH led to smaller particles. SNS did not lead to hemolysis or platelet activation and had no significant effect on coagulation or fibrinolysis. Animal experiments suggested that SNS did not affect blood cell counts or hepatorenal function. In cell experiments, SNS increased LDH release and intracellular ROS, which is consistent with intracellular protein assays. An immune compatibility assay found that SNS did not induce the release of IL-1*β* from macrophages, but did induce the release of TGF-*β*1. In conclusion, SNS appeared to have good biosafety and antitumor activities, supporting its potential as an anticancer drug for clinical use.

## Figures and Tables

**Table 1:** The effects of NPs on coagulation test results.

Groups	PT (s)	APTT (s)	TT (s)	cFIB (g/L)
NC	10.33 ± 0.17	25.60 ± 0.56	18.40 ± 0.68	2.79 ± 0.55
PC	9.40 ± 0.14	11.40 ± 0.08^∗∗^	17.05 ± 2.38	1.65 ± 0.42^∗^
SNS without anti-PD-L1	10.55 ± 0.13	27.45 ± 0.52	20.20 ± 0.25	2.68 ± 0.08
SNS without Nano-Ag	10.35 ± 0.13	27.95 ± 0.41	19.08 ± 0.99	2.66 ± 0.15
SNS without Dox	10.48 ± 0.10	28.45 ± 1.37	17.78 ± 0.30	2.63 ± 0.10
SNS without MnO_2_	10.38 ± 0.10	28.05 ± 0.52	18.00 ± 0.35	2.62 ± 0.06
SNS	10.65 ± 0.06	28.00 ± 0.47	18.65 ± 0.42	2.64 ± 0.01

Note: ^∗^ and ^∗∗^, indicate that, compared with NC, *p* < 0.05 and *p* < 0.01, respectively.

**Table 2:** The effects of NPs on blood cells and biochemical parameters.

Groups	WBC (×10^9^/L)	HB (g/L)	PLT (×10^9^/L)	ALT (IU/L)	AST (IU/L)	BUN (mmol/L)	Scr (*μ*mol/L)
NC	7.36 ± 1.54	143.15 ± 18.97	462.35 ± 42.78	52.47 ± 7.84	43.86 ± 5.79	13.42 ± 2.81	124.68 ± 14.57
PC	3.14 ± 1.98^∗∗^	97.07 ± 16.48^∗^	231.92 ± 37.54^∗∗^	132.59 ± 15.88^∗∗^	87.63 ± 11.74^∗∗^	35.81 ± 9.06^∗∗^	189.82 ± 33.28^∗^
SNS without anti-PD-L1	8.06 ± 2.19	126.08 ± 24.63	407.11 ± 64.55	48.69 ± 5.72	38.61 ± 8.71	16.44 ± 2.57	138.44 ± 23.61
SNS without Nano-Ag	6.93 ± 1.72	157.03 ± 19.46	459.32 ± 34.03	57.53 ± 4.88	41.56 ± 3.89	12.69 ± 3.78	114.65 ± 17.56
SNS without Dox	7.91 ± 2.41	135.39 ± 14.16	506.16 ± 89.97	46.05 ± 3.21	44.75 ± 6.09	15.06 ± 2.14	146.37 ± 19.72
SNS without MnO_2_	6.74 ± 2.83	161.54 ± 28.91	531.06 ± 79.58	51.06 ± 6.54	39.56 ± 4.88	17.01 ± 3.22	109.81 ± 22.43
SNS	8.25 ± 1.79	149.44 ± 12.09	493.05 ± 67.52	56.43 ± 5.01	46.77 ± 5.16	11.76 ± 3.08	133.66 ± 26.78

Note: ^∗^ and ^∗∗^ indicated that compared with NC, *p* < 0.05 and *p* < 0.01, respectively.

## Data Availability

Any display item and related data are available upon request.
